# The Awareness of Child Abuse and Neglect Among the Saudi Population: A Narrative Review

**DOI:** 10.7759/cureus.49894

**Published:** 2023-12-04

**Authors:** Nahi Sabih Q Alruwaili, Abdulaziz Mohammed M Alanazi, Naif M Alrawaili, ‏Abdullah Khalid M Alzalbani, ‏Malek Saad M Alanazi, ‏Fahad Abdullah J Alotibi, ‏Rakan Zuwayyid A Alanazi, Mooj Mohammed Alruwaili, Abdulrahman Mohammed M Alanzi

**Affiliations:** 1 Psychiatry, Eradah Complex and Mental Health, Arar, SAU; 2 Medicine, Eradah Complex and Mental Health, Arar, SAU; 3 General Practice, Ministry of Health, Arar, SAU; 4 General Practice, Northern Border University, Arar, SAU; 5 Pediatrics, Northern Border University, Arar, SAU; 6 Medicine, Northern Border University, Arar, SAU

**Keywords:** saudi population, attitudes, knowledge, awareness, child neglect, child abuse

## Abstract

Child abuse and neglect (CAN) is a pressing global issue with profound implications for the well-being of children. The aim of this review is to examine the existing literature and synthesize evidence on the awareness, knowledge, and attitudes toward child abuse and neglect within the Saudi population. This review synthesizes the existing literature to illuminate the awareness, knowledge, and attitudes surrounding CAN within the Saudi population. Ten studies spanning the years 2019-2023 were meticulously analyzed, offering a comprehensive snapshot of CAN perceptions across various segments of society. The studies, encompassing diverse methodologies and populations, collectively underscore the commendable levels of awareness and knowledge demonstrated by healthcare professionals, including primary healthcare (PHC) physicians, nurses, and medical practitioners. Notably, several studies reveal that these professionals exhibit robust recognition of different forms of child abuse, a vital aspect in the identification and prevention of abuse cases. While positive attitudes toward CAN were prevalent among healthcare providers, barriers to reporting were highlighted. The fear of consequences and uncertainty emerged as key deterrents to reporting among medical and dental students and nurses, respectively. These findings emphasize the necessity for creating supportive environments that empower professionals to report suspected cases of abuse while addressing apprehensions. The parental perception of CAN also came into focus, revealing a disparity between the recognition of CAN as a common problem and the adequate knowledge of emotional abuse and neglect. These findings point toward a need for targeted public awareness campaigns that differentiate between disciplinary practices and abusive behaviors. In conclusion, this review offers a nuanced understanding of the Saudi population's awareness, knowledge, and attitudes toward child abuse and neglect. The synthesis of findings across diverse studies informs future interventions, advocating for enhanced awareness, reporting, and prevention strategies. By empowering individuals and professionals alike, a safer environment for Saudi children can be nurtured, fostering a future free from the shadows of abuse and neglect.

## Introduction and background

Child abuse and neglect (CAN) are globally recognized as significant public health issues with profound and lasting consequences for the physical, psychological, and social well-being of children [1]. CAN encompasses various forms, including physical, sexual, and emotional abuse and neglect, and can occur in diverse settings such as households, schools, and communities [2]. While the detrimental effects of CAN are well-documented, its prevalence and impact can vary across cultures and regions [1].

Saudi Arabia, as a diverse and culturally rich country, is not exempt from the challenges posed by CAN. The societal values, norms, and dynamics unique to Saudi Arabia may influence perceptions, awareness, knowledge, and attitudes toward CAN [3]. Addressing CAN requires a comprehensive understanding of these factors, as well as an assessment of the existing awareness, knowledge, and attitudes within the Saudi population [4].

Given the significance of this issue, it is imperative to examine the current landscape of CAN awareness, knowledge, and attitudes among different segments of the Saudi population. Such insights can guide the development of targeted interventions, policies, and educational programs to promote greater awareness, accurate recognition, and effective response to cases of child abuse and neglect. The primary aim of this review is to comprehensively examine the existing literature and synthesize the collective evidence on the awareness, knowledge, and attitudes toward child abuse and neglect within the Saudi population.

## Review

A narrative review protocol was developed to guide the entire review process. The research question formulated was, "What is the awareness, knowledge, and attitude of the Saudi population toward child abuse and neglect (CAN) based on studies conducted in Saudi Arabia over the last five years?"

A comprehensive literature search was conducted across multiple electronic databases, including PubMed, Scopus, Web of Science, and PsycINFO. The search was limited to studies published within the last five years (2019-2023). The search strategy employed a combination of keywords and Medical Subject Heading (MeSH) terms related to child abuse, neglect, Saudi Arabia, awareness, knowledge, and attitude. Boolean operators (AND and OR) were used to refine the search results.

Studies were selected based on predefined inclusion and exclusion criteria. The inclusion criteria encompassed studies conducted in Saudi Arabia and published in the English language, between 2019 and 2023, that examined awareness, knowledge, and attitudes toward child abuse and neglect among the Saudi population. The exclusion criteria included nonempirical studies, studies conducted outside Saudi Arabia, and those not related to the research question.

The initial screening of studies involved a title and abstract review, followed by a full-text assessment to determine eligibility. Two independent reviewers conducted the screening process, with disagreements resolved through discussion and consensus. Data extraction was carried out using a standardized form that captured information including study title; year; location; study design; study setting; type of population; number of participants; study tool description; summary findings related to child abuse awareness, knowledge, and attitude; and any notable observations. The findings from individual studies were summarized, compared, and contrasted to draw overarching conclusions regarding awareness, knowledge, and attitudes toward child abuse and neglect in the Saudi population.

Results

A total of 272 study articles resulted from the search, and 224 were automatically removed. Title and abstract screening was conducted on 72 studies, and 26 studies were excluded. Forty-six studies were sought for retrieval, and only 19 articles were retrieved. Finally, 19 studies were screened for full-text assessment; nine studies were excluded for having either inappropriate study methodology or results. Ten eligible study articles were included in this review.

Table 1 provides an overview of the characteristics of the included studies in this review. The selected studies were conducted across different years and cities within Saudi Arabia, employing various study designs and settings to investigate the awareness, knowledge, and attitudes toward child abuse and neglect (CAN) among different populations.

**Table 1:** Characters of the included studies. UG, undergraduate; CAN, child abuse and neglect

Study	Location	Study Design	Study Setting	Type of Population	Number of Population	Study Tool Description	Summary Findings About Child Abuse Awareness
Alsaleem et al., 2019 [5]	Abha	Cross-sectional	Primary healthcare (PHC)	PHC physicians	300	Self-administered questionnaire	About 96.3% had a good awareness of types of child abuse; 97.3% had a good awareness of child neglect patterns. About 64% recorded underreporting of child abuse cases.
Gopalakrishna et al., 2020 [6]	Riyadh	Cross-sectional	Medical and dental	Medical and dental UG students and interns	351	Self-administered structured questionnaire	The mean knowledge scores were 6.81 for medical and 6.35 for dental students (significant difference). The fear of consequences was the main barrier for not reporting (medical = 82.4%; dental = 68.5%). About 77% agreed on the need for further training.
Sulimany et al., 2021 [7]	Nationwide	National study	Dental graduates	Dental graduates	988	Self-administered questionnaires	About 60% had inadequate knowledge regarding CAN. Education about CAN, female participants, and government school graduates were associated with better knowledge scores. Only 9.7% knew how to report CAN cases.
Alsalman et al., 2023 [8]	Eastern Province	Cross-sectional	Parents	Parents with children <18 years old	447	Self-administered online questionnaire	Only 53% had good knowledge of CAN. Higher education and family income levels were associated with good knowledge. Most parents considered CAN a common problem but lacked knowledge of emotional abuse and neglect.
Alkathiri et al., 2021 [9]	Riyadh	Cross-sectional	Healthcare providers	Physicians and nurses	308	Self-administered questionnaire	Majority had excellent attitude (64.2%) and excellent knowledge (90.3%). Pediatricians had lower knowledge (p < 0.001). The lack of knowledge was a common reason for underreporting (67.2%).
Alharbi and Moussa, 2023 [10]	Riyadh	Quantitative	Pediatric nurses	Pediatric nurses	70	Self-administered questionnaire	Pediatric nurses had adequate knowledge and positive attitudes toward child abuse. Experience and middle age were associated with better knowledge and attitude, respectively.
Aldukhayel et al., 2020 [11]	Al Qassim	Cross-sectional	Primary healthcare	Primary healthcare physicians and interns	292	Self-administered questionnaire	Majority had moderate (68.8%) to high (28.4%) knowledge levels. Factors associated with high knowledge included age of >30, non-Saudi nationality, having children, and studying outside Saudi Arabia. Pediatricians had lower knowledge (p < 0.001).
Alshouimi et al., 2021 [12]	Saudi Arabia	Cross-sectional	Medical students	Medical students	403	Cross-sectional questionnaire	Good knowledge about risk factors and symptoms but with the lack of confidence. The majority believed that further curriculum development and training are necessary.
Merwass et al., 2021 [13]	Saudi Arabia	Cross-sectional	Medical and dental	Medical and dental practitioners	371	Online self-administered questionnaire	Good knowledge (mean score: 7.51). Females and governmental practitioners had higher knowledge. Training needed for dealing with CAN (65.2%). Majority believed that uncertainty was the prime reason for underreporting.
Salami and Alhalal, 2020 [14]	Riyadh	Cross-sectional	Nurses	Nurses in emergency and pediatric units	248	Self-administered questionnaires	Knowledge, subjective norms, and organizational support were associated with intention to report child abuse. Nurses reported higher intention to report child sexual abuse. Clinical practice areas influenced reporting intention.

The studies were conducted between 2019 and 2023 in various cities across Saudi Arabia. A range of study designs were employed, including cross-sectional and quantitative approaches, allowing for a comprehensive examination of the awareness, knowledge, and attitudes of diverse populations. The study settings varied, including primary healthcare (PHC) centers, medical and dental colleges, and pediatric units, reflecting the multidisciplinary nature of the investigation into CAN.

The studies targeted different segments of the population, such as primary healthcare physicians, medical and dental undergraduate students, parents, pediatric nurses, and other healthcare providers. The number of participants ranged from 70 to 988, ensuring a robust representation of the chosen populations and increasing the generalizability of the findings.

To assess awareness, knowledge, and attitudes toward child abuse and neglect, the included studies utilized various self-administered questionnaires and structured tools. These tools were designed to capture the participants' perceptions, understanding, and attitudes related to different aspects of child abuse, providing insights into the knowledge gaps and attitudes prevalent within the studied populations.

The summary findings from the included studies offer insights into the participants' awareness, knowledge, and attitudes concerning child abuse and neglect. The findings collectively demonstrate a spectrum of understanding and attitudes within different segments of the population.

The study by Alsaleem et al. (2019) [5] assessed primary healthcare (PHC) physicians' knowledge and attitudes toward child abuse in Abha. The findings revealed that 96.3% of the physicians exhibited good awareness of the types of child abuse, and 97.3% demonstrated good awareness of child neglect patterns. However, a concerning 64% of physicians recorded underreporting of child abuse cases, indicating the presence of barriers hindering the reporting process. Gopalakrishna et al. (2020) [6] conducted a cross-sectional study among medical and dental undergraduate students and interns in Riyadh. The results highlighted that approximately 57.5% of participants had received formal training on CAN during their undergraduate studies. Medical participants displayed a higher mean knowledge score (6.81) compared to dental participants (6.35). The fear of consequences emerged as a predominant barrier (medical = 82.4%; dental = 68.5%) for not reporting suspected cases of CAN. Sulimany et al. (2021) [7] conducted a national study to assess knowledge levels and educational experiences among dental graduates regarding child abuse and neglect. The study revealed that around 60% of participants had inadequate knowledge regarding CAN. Notably, graduates from government schools who received dental education about CAN and female participants demonstrated significantly higher odds of having adequate knowledge scores.

Alsalman et al. (2023) [8] investigated parents' knowledge and perception of child abuse and neglect in the Eastern Province. Only 53% of the participants demonstrated good knowledge of CAN. Higher education and family income levels were associated with better knowledge. While most parents considered CAN to be a common problem, many lacked knowledge about emotional abuse and neglect. Alkathiri et al. (2021) [9] assessed the knowledge, attitude, and practice of child maltreatment among healthcare providers in Riyadh. A majority of participants (64.2%) displayed excellent attitudes, and an even higher percentage (90.3%) exhibited excellent knowledge. Pediatricians, however, had lower knowledge scores. The lack of knowledge emerged as a common barrier to reporting (67.2%). Alharbi and Moussa (2023) [10] investigated the knowledge and attitude of pediatric nurses in Saudi Arabia regarding child abuse. The study found that pediatric nurses displayed adequate knowledge and a positive attitudes toward child abuse. Age and experience were positively associated with knowledge and attitude.

Aldukhayel et al. (2020) [11] examined the knowledge and attitude of primary healthcare physicians and interns in Al Qassim. A majority had moderate (68.8%) to high (28.4%) knowledge levels. Pediatricians exhibited lower knowledge scores. The lack of knowledge was a common reason for underreporting (67.2%). Alshouimi et al. (2021) [12] conducted a study among medical students in Saudi Arabia. The results indicated good knowledge about risk factors and symptoms of child abuse but a lack of confidence. Participants highlighted the need for curriculum development and further training in this area. Merwass et al. (2021) [13] assessed knowledge and attitudes toward child abuse and neglect among dental and medical practitioners. Most participants demonstrated good knowledge (mean score: 7.51) regarding CAN. Training needs were identified for dealing with CAN cases. Uncertainty emerged as a prime reason for underreporting. Salami and Alhalal (2020) [14] explored nurses' intention to report child abuse in Saudi Arabia. Factors such as knowledge, subjective norms, and organizational support were associated with nurses' intention to report. Clinical practice areas influenced reporting intentions, with nurses reporting a higher intention to report child sexual abuse.

Discussion

The present review sought to shed light on the awareness, knowledge, and attitudes of the Saudi population toward child abuse and neglect (CAN), as gleaned from a diverse array of studies conducted in Saudi Arabia. The findings synthesized from these studies offer a nuanced understanding of the complex landscape surrounding CAN awareness, uncovering both strengths and gaps in different segments of the population.

Across the studies, it is heartening to note that many participants, particularly healthcare professionals, displayed commendable levels of knowledge concerning the various facets of child abuse. The findings from Alsaleem et al. [5], Alkathiri et al. [9], and Merwass et al. [13] collectively indicate that healthcare providers, including primary healthcare physicians, nurses, and even medical and dental practitioners, exhibited robust awareness of the different forms of child abuse. This knowledge is pivotal in the identification, intervention, and prevention of CAN cases.

However, a caveat emerges from the study by Sulimany et al. [7], which points to a significant gap in the knowledge of dental graduates regarding CAN. This divergence highlights the need for a more uniform and comprehensive approach to CAN education across medical and dental curricula, ensuring that future practitioners are adequately equipped to address this critical issue.

Attitudes toward CAN appeared to be generally positive among the populations surveyed. The studies conducted by Alkathiri et al. [9], Alharbi and Moussa [10], and Merwass et al. [13] demonstrated that healthcare professionals, including nurses and healthcare providers, exhibited positive attitudes toward CAN. Notably, pediatric nurses emerged as particularly proactive in recognizing their role in identifying and addressing cases of child abuse.

Interestingly, some studies unearthed barriers to reporting CAN cases, which warrant careful consideration. Gopalakrishna et al. [6] indicated that the fear of consequences was a significant deterrent among medical and dental students, pointing to a potential apprehension among future healthcare providers. Similarly, Salami and Alhalal [14] found that uncertainty was a major reason for underreporting among nurses. These findings underscore the importance of creating a supportive reporting environment, coupled with a comprehensive education that emphasizes the legal and ethical implications of reporting suspected cases of abuse.

The studies delving into parental awareness unveiled intriguing insights. Alsalman et al. [8] revealed that while parents perceived CAN as a common problem, a sizable proportion lacked adequate knowledge of the nuances of emotional abuse and neglect. This indicates a vital gap in public awareness that necessitates targeted awareness campaigns, focusing on differentiating between traditional discipline and abusive behavior [15].

Collectively, the findings carry significant implications for policy, education, and practice. The identified gaps in knowledge, coupled with reported barriers to reporting, underscore the need for a comprehensive and standardized educational approach across various healthcare disciplines. The curricula should integrate evidence-based education on identifying and responding to CAN cases, addressing fears and uncertainties that could hinder reporting.

Public awareness campaigns can play a pivotal role in enhancing the societal understanding of CAN, not only among healthcare professionals but also among parents and the general public. As observed in the study by Alsaleem et al. (2019) [5], societal traditions and unclear reporting strategies could contribute to underreporting, highlighting the importance of cultural sensitivity in developing interventions.

## Conclusions

This review amalgamates findings from various studies to present a multifaceted overview of the awareness, knowledge, and attitudes toward child abuse and neglect among different segments of the Saudi population. While the healthcare community displays commendable awareness and attitudes, there is still room for improvement, particularly in standardizing education and addressing barriers to reporting. The insights garnered from this review can guide future research to explore some of these variables in a specific group, fostering a safer environment in Saudi Arabia by empowering individuals to recognize, report, and prevent child abuse and neglect.
